# Priapism associated with penile haematoma following a scorpion sting in a child: A rare case report

**DOI:** 10.1016/j.eucr.2023.102508

**Published:** 2023-07-19

**Authors:** Omar Bellouki, Hicham EL Bote, Jihad Lakssir, Abdelmounim Boughaleb, Youssef Abaair

**Affiliations:** a Department of Urology A, Ibn Sina University Hospital of Rabat, Morocco; b Department of Surgery, Regional Hospital Center of Beni Mellal, Morocco

**Keywords:** Priapism, Penile haematoma, Scorpion sting, Envenomation

## Abstract

Priapism is a rare condition in paediatrics. Although its association with scorpion envenomation has been documented, cases involving an associated penile haematoma are extremely rare. To the best of our knowledge, we hereby present the first documented case of this unique association in a nine-year-old boy following a scorpion sting. The purpose of this observation is to discuss the diagnostic difficulties, management strategies, and possible mechanisms associated with this unusual manifestation, and to highlight the importance of prompt recognition and appropriate management of priapism and penile haematoma in children, particularly those living in areas where scorpion stings are endemic.

## 1 Introduction

Although priapism has been reported as a complication of scorpion envenomation in adults, its occurrence in paediatrics is uncommon.^1^ The development of a penile haematoma associated with priapism in this context is exceedingly rare.

Scorpionism is a serious public health issue worldwide, particularly in regions with a high prevalence of these arachnids, such as Morocco.^2^ We present a unique case of a nine-year-old boy who was the victim of scorpion sting, which resulted in priapism, later complicated by the development of an unusual penile haematoma.

## 2 Case report

A 9-year-old boy was brought to the emergency department (ED) 12 hours after being stung on his left foot by a yellowish scorpion identified as *Buthus occitanus*, which belongs to the Buthidae family.

The child's parents initially opted for natural treatments due to the distance between their residence and the hospital. However, as the child's symptoms persisted, the parents recognized the need for medical intervention.

**Fig. 1:**
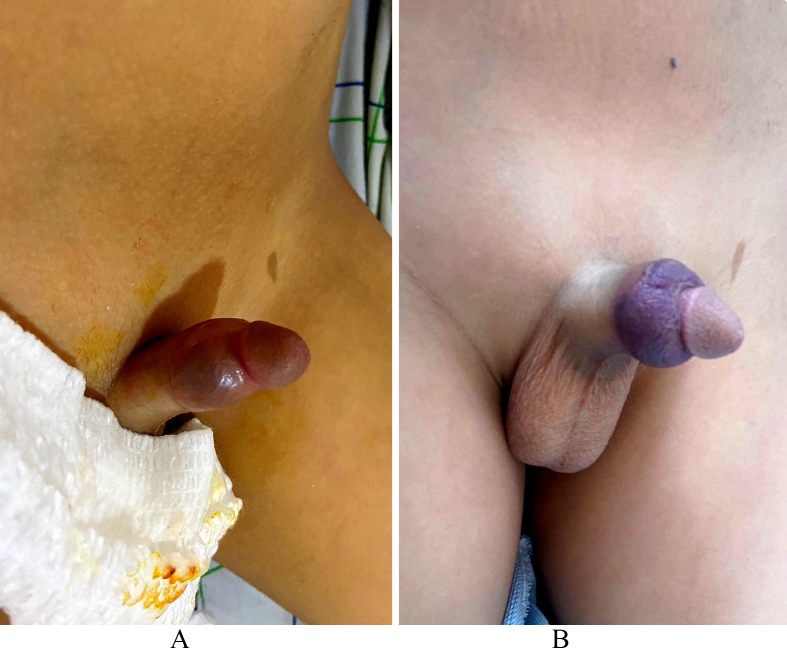
Images showing the different clinical stages of the penis. A: Priapism, B: Penile haematoma.

Upon arrival to the ED, the child exhibited localized pain and tenderness at the site of the scorpion sting. Blood pressure measurements showed a spike, with a reading of 170/110 mm Hg. The most significant finding was the presence of persistent priapism, causing discomfort and pain in the penis (Fig. 1A). The erection occurred shortly after the sting, without any context of genital stimulation or trauma.

The patient was admitted to the intensive care unit (ICU), where continuous blood pressure monitoring was initiated and intravenous nicardipine was administered. Afterwards, the patient presented a severe hypotension, necessitating the use of dobutamine. Conservative management of the priapism was decided, involving the application of ice packs and cold compresses to the penile area, alongside intracavernous injections of ephedrine. Pain management was assured using non opioid analgesics.

The priapism gradually resolved, and detumescence was achieved shortly thereafter. Close monitoring was maintained to ensure absence of recurrence. The patient's hemodynamic state was restored. He remained in the ICU for 4 days under observation.

**Fig. 2:**
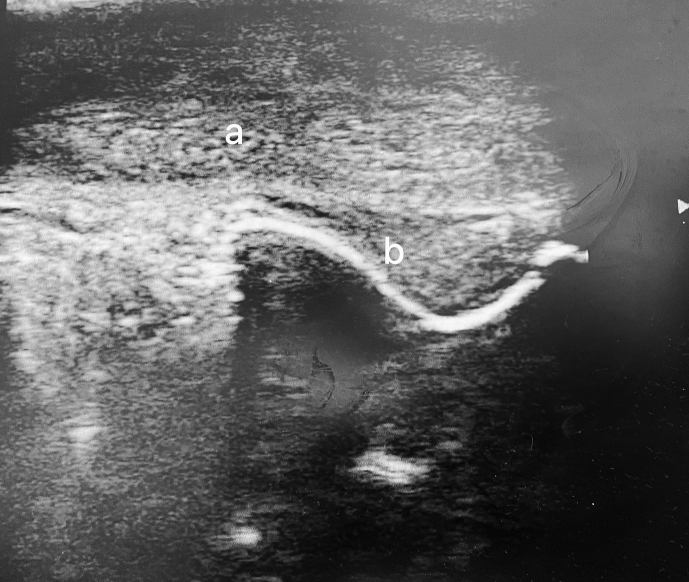
Penile ultrasound showing the corpus cavernosum (a) and the haematoma (b).

During the follow-up period, we noticed the occurrence of a penile haematoma regarding the retroglandular sulcus (Fig. 1B). Ultrasound confirmed it as a subcutaneous haematoma with no signs of active bleeding (Fig. 2). Despite thorough investigations, no definitive cause for the haematoma could be identified.

**Fig. 3:**
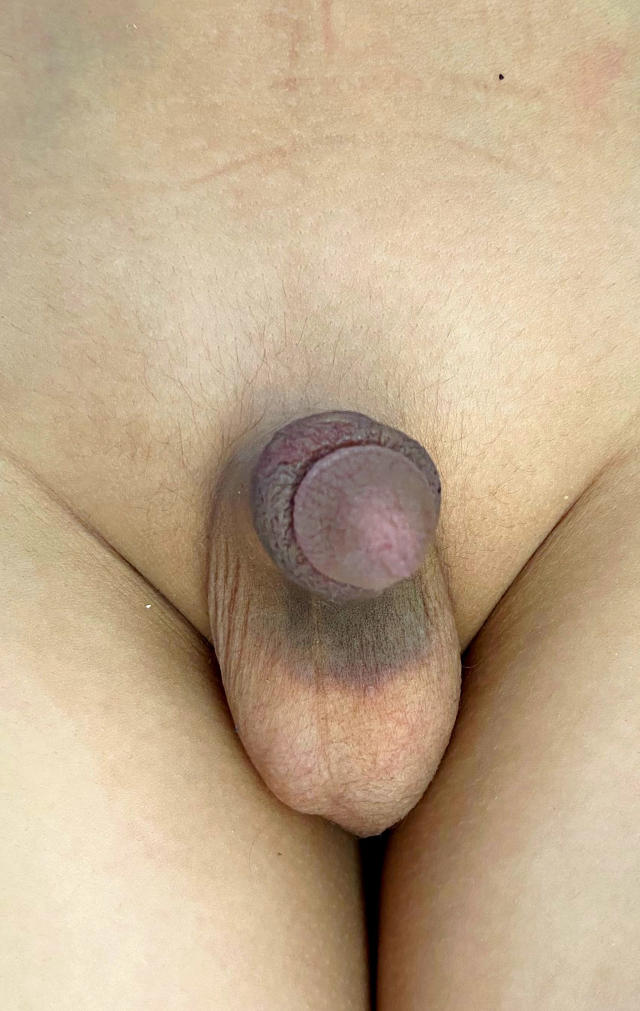
Residual penile ecchymosis.

Same initial conservative measures were maintained. Over the course of six days, the haematoma gradually decreased, leaving a simple ecchymosis (Fig. 3).

## 3 Discussion

*Buthus occitanus* species can cause severe envenomation representing a critical medical emergency, particularly among children, due their immature defense mechanisms and the correlation between venom dosage and body weight.^3^ It is a significant public health concern in North Africa, including Morocco, where the Moroccan poison control and pharmacovigilance center (CAPM) records approximately 30,000 cases of scorpion sting annually, with 25% affecting children under the age of 15.^2^

Clinical presentation of scorpion envenomation is diverse and classified into three categories: Class 1 includes asymptomatic patients or those presenting localized signs, Class 2 involves minor systemic signs (not life-threatening), and Class 3 represents life-threatening distress with one or more signs of vital failure. Priapism is recognized as an indicative factor of severity,^3^ requiring admission to an ICU.

Priapism can be classified into two types: ischemic (low-flow) and non-ischemic (high-flow) priapism. It is crucial to differentiate between these types as ischemic priapism can lead to complications such as penile fibrosis and erectile dysfunction. Another presentation, known as stuttering priapism, has been identified as a distinct third type. It is characterized by multiple episodes of painful erections occurring during sleep. Scorpion stings typically result in the development of ischemic priapism.^1^

The occurrence of priapism involves the activation of autonomic and sympathetic nervous system pathways. Scorpion venom stimulates the release of neurotransmitters and neuropeptides, including nitric oxide (NO), which influences vascular tone and smooth muscle relaxation in the penile erectile tissue. Nitric oxide plays a vital role in regulating penile blood flow and inducing vasodilation. Excessive release of NO in can result in prolonged and uncontrolled vasodilation, leading to a persistent erection.^4^

Diagnosis of priapism relies on clinical assessment, as observed in our case. Additional tests as cavernous blood gas analysis, haemogram, platelet count, and coagulation panel may be conducted as part of the etiological investigation. Doppler ultrasound is useful for non-ischemic priapism to locate the fistula site, while MRI of the penis can be considered to assess the viability of penile muscles.^1^

Priapism is managed gradually, starting with penile aspiration and intracavernous injection of alpha-adrenergic agonists. Surgical shunts may be considered in the case of failure of these measures for ischemic priapism. However, non-invasive treatments like local cooling, massages, physical activity, and administration of oral alpha-stimulants can also be proposed.^1^ In our case, conservative treatment was successful as detumescence was promptly obtained.

The management of associated scorpion envenomation follows a multimodal approach. The use of antivenom remains a topic of debate and controversy due to the lack of conclusive evidence regarding its efficacy, its limited cost-effectiveness, and the potential risk of anaphylactic and pyrogenic reactions.^5^ In Morocco, the therapeutic protocol is based on symptomatic treatment. In severe clinical presentations (Class 2 with severity factors or Class 3), admission to the ICU is required for close monitoring and supportive treatments.^2^

Follow-up is essential for detecting recurrence and potential complications. In our case, a rare complication occurred, manifesting as a penile haematoma in the retroglandular sulcus, with no other symptoms. Our medical team supposed that this complication might be related to a self-inflicted trauma, considering the child's agitation during their ICU stay.

## 4 Conclusion

Priapism and subsequent penile haematoma resulting from scorpion envenomation in paediatric patients are rare occurrences. Prompt recognition and appropriate management are crucial in ensuring favorable outcomes, particularly in high-prevalence regions. Medical awareness, timely intervention, and access to medical facilities are vital for patients residing in remote areas. In addition, psychological support for children in these situations is imperative to reduce possible and unexpected complications, as observed in our case.

## Author contribution

All authors have contributed to this work and have read and approved the final version of the manuscript.

## Declaration of competing interest

The authors declare no conflict of interest.
